# Comparison of clinical severity and epidemiological spectrum between coronavirus disease 2019 and influenza in children

**DOI:** 10.1038/s41598-021-85340-0

**Published:** 2021-03-11

**Authors:** Maria Pokorska-Śpiewak, Ewa Talarek, Jolanta Popielska, Karolina Nowicka, Agnieszka Ołdakowska, Konrad Zawadka, Barbara Kowalik-Mikołajewska, Anna Tomasik, Anna Dobrzeniecka, Marta Lipińska, Beata Krynicka-Czech, Urszula Coupland, Aleksandra Stańska-Perka, Małgorzata Ludek, Magdalena Marczyńska

**Affiliations:** 1 Department of Children’s Infectious Diseases, Medical University of Warsaw, Warsaw, Poland; 2 Regional Hospital of Infectious Diseases in Warsaw, Warsaw, Poland; 3 Medical University of Warsaw, Warsaw, Poland

**Keywords:** Health care, Medical research

## Abstract

Data on the novel coronavirus disease 2019 (COVID-19) in children are limited, and studies from Europe are scarce. We analyzed the clinical severity and epidemiologic aspects of COVID-19 in consecutive children aged 0–18 years, referred with a suspicion of COVID-19 between February 1, and April 15, 2020. RT-PCR on a nasopharyngeal swab was used to confirm COVID-19. 319 children met the criteria of a suspected case. COVID-19 was diagnosed in 15/319 (4.7%) patients (8 male; mean age 10.5 years). All of them had household contact with an infected relative. Five (33.3%) patients were asymptomatic. In 9/15 (60.0%) children, the course of the disease was mild, and in 1/15 (6.7%), it was moderate, with the following symptoms: fever (46.7%), cough (40%), diarrhea (20%), vomiting (13.3%), rhinitis (6.7%), and shortness of breath (6.7%). In the COVID-19-negative patients, other infections were confirmed, including influenza in 32/319 (10%). The clinical course of COVID-19 and influenza differed significantly based on the clinical presentation. In conclusion, the clinical course of COVID-19 in children is usually mild or asymptomatic. In children suspected of having COVID-19, other infections should not be overlooked. The main risk factor for COVID-19 in children is household contact with an infected relative.

## Introduction

The 2019 novel severe acute respiratory syndrome coronavirus (SARS-CoV-2) is currently causing an outbreak of coronavirus disease 2019 (COVID-19), which is an emerging global threat that is rapidly spreading throughout the world^1,2^. On March 11, 2020, the World Health Organization (WHO) declared the outbreak of SARS-CoV-2 a pandemic^3^. In April 2020, the epicenter of the pandemic moved from China to the United States (US) and Europe.

Data on SARS-CoV-2 infection in children are scarce^1,2,4^. Children seem to be less likely to be affected by the disease. Among over 72,000 COVID-19 cases from China, only 1.2% of the patients were children 10 to 19 years of age, and 0.9% were children under 10 years of age^5^. Pediatric cases of COVID-19 outside China are reported sporadically. According to the available data, the proportion of children among all infected patients ranged between 0.6 and 2.4% in China, 0.8% in Spain, 1.4% in Italy, 1.7% in the US, 2.8% in Australia, 3.6% in Singapore, and 1.0–5.2% in the Republic of Korea^4,6,7^. The clinical course of COVID-19 in children seems to be less severe than that in adults, with fewer clinical symptoms and case-fatality rates close to 0%^1,2^. In the study by Wu and McGoogan, involving over 72,000 COVID-19 cases in China, with an overall case-fatality rate of 2.3%, only one death occurred in an adolescent, and no child younger than 10 years of age died^5^.

There is also evidence suggesting that children are as likely as adults to become infected, but they are less likely to develop severe clinical symptoms or any symptoms at all^1^. Several observations suggest that children may experience different clinical symptoms than adults^7–9^. In addition, asymptomatic or mildly symptomatic children may transmit the disease, posing a risk of infection to their adult and elderly relatives^10^.

As data on COVID-19 in children are limited and studies in this field from the European region are scarce, we aimed to analyze the clinical and epidemiologic aspects of COVID-19 in children. In particular, we have focused on the role of the epidemiological and clinical features for screening SARS-CoV-2 infection and the severity of COVID-19 in pediatric patients. In addition, we aimed to compare the clinical course of COVID-19 with COVID-19-negative patients diagnosed with other illnesses, including influenza.

## Materials and methods

All consecutive pediatric patients aged 0–18 years referred to our tertiary health care department between February 1, 2020, and April 15, 2020 with a suspicion of COVID-19 were included in this prospective observational study. The suspected cases presented as having clinical symptoms of the disease or a positive epidemiological history (international travel or contact with an infected person). Household contact was defined as living with a relative positively tested for SARS-CoV-2 infection, irrespective of the clinical presentation. The Regional Hospital of Infectious Diseases in Warsaw is the main center dedicated to COVID-19 patients in central Poland and sees patients of all ages. To our knowledge, about 25% of all children with COVID-19 in Poland are seen in our center. The first COVID-19 case in Poland was diagnosed on March 4, 2020, and until April 17, 2020, there were 8,379 confirmed cases, and 332 deaths were reported. Our region in central Poland is the most affected, with 1883 cases (348.5 cases per million). Data on the ages of the positively diagnosed patients are unavailable.

For clinical purposes and qualifying patients for testing for SARS-CoV-2 infection, the case definition from the WHO with its amendments was used, which also formed the basis for the definition of a case suspected for COVID-19 from the European Centre for Disease Prevention and Control (ECDC)^1,11^. Thus, before local transmission in Poland occurred (March 11, 2020), there were patients referred to our department who did not meet the criteria of a suspected case. These patients were not tested for COVID-19 and were excluded from the final analysis. Demographical, epidemiological, and clinical data were analyzed, and cases were grouped based on the results of COVID-19 testing. In addition, the clinical presentations of COVID-19 and influenza were compared.

The clinical course of COVID-19 was defined as follows: asymptomatic, when no complaints or symptoms were present at or before the moment of diagnosis and no abnormalities were found on physical examination; mild, when symptoms of upper respiratory tract infection were present, with or without fever and other complaints (e.g., fatigue, myalgia), and without pneumonia (either on auscultation or chest X-ray); moderate, when pneumonia (confirmed by chest X-ray) but no hypoxemia was present; severe, when pneumonia (confirmed by chest X-ray) progressing to dyspnea with oxygen saturation < 92% was present; and critical, when acute respiratory distress syndrome, shock, or any organ failure occurred. Similar definitions have been used by other authors^9,12^.

For the diagnosis of SARS-CoV-2 infection, real-time polymerase chain reaction (RT-PCR) on a nasopharyngeal swab was performed in a certified molecular diagnostics laboratory using a certified method (COVID-19 Genesig Real-Time PCR Assay, Primerdesign Ltd., Chandler’s Ford, UK)^13^. Infection was defined as at least one positive test result. To establish the diagnosis in children negative for SARS-CoV-2, further laboratory and microbiological testing was performed based on the clinical presentation. To diagnosis influenza, we used commercially available rapid influenza diagnostic tests. In case of a negative result in children suspected for influenza or respiratory syncytial virus (RSV) infection, a PCR method was used (Cepheid Xpert Xpress Flu/RSV, Maurens-Scopont, France). To confirm group A Streptococcal infection, commercially available Rapid Strep Tests were used.

### Statistical analysis

Data were assessed for normal distribution using the Kolmogorov–Smirnov test. Continuous variables were presented as medians with interquartile ranges (IQRs) and were compared using the Mann–Whitney test. Categorical variables were compared using either the chi-square test or Fisher’s exact test, as appropriate. A two-sided p value of < 0.05 was considered significant. All statistical analyses were performed using MedCalc Statistical Software version 19.1.1 (MedCalc, Ostend, Belgium, https://www.medcalc.org).

### Ethical statement

The investigation was performed in accordance with the ethical standards in the 1964 Declaration of Helsinki and its later amendments. The local Ethics committee by the Regional Medical Chamber in Warsaw approved this study and the treatment protocol for pediatric patients with COVID-19. Written informed consent was collected from all the patients and/or their parents/guardians before their inclusion in the study.

## Results

### Study population

During the period analyzed, 423 patients were referred to our department with suspicion of COVID-19. Of them, 104 did not meet the current WHO definition of a case suspected for COVID-19 (before the local transmission occurred in Poland). They were not qualified for testing for SARS-CoV-2 infection and were excluded from the study. Thus, 319 patients, aged 14 days to 18 years, were tested for SARS-CoV-2 infection with the PCR method and were included in the final analysis. Almost a quarter of the patients had a history of international travel in the 14 days before admission (most commonly to Italy), and 13.5% had household contact with a relative with documented COVID-19. The demographic and epidemiological characteristics of the study group are presented in Table 1.

**Table 1:** Demographic, epidemiological, and clinical characteristics of the study group.

Characteristics	Total	COVID-19	COVID-19	Influenza	*P*	*P*
		Positive	Negative	Positive	COVID-19 positive vs. COVID-19 negative	COVID-19 positive vs. influenza positive*
Number of patients	319	15	304	32		
*Demographic and epidemiological features*						
Sex						
Male /Female	162 (50.8) / 157 (49.2)	8 (53.3) / 7 (46.7)	154 (50.7)/ 150 (49.3)	19 (59.3) / 13 (40.7)	0.74	0.69
Age (months)						
Median (IQR)	88 (36; 145)	128 (77.25; 140)	84 (35.5; 145.5)	112 (79.5; 162)	0.37	0.72
Duration of clinical symptoms before admission (days); Median (IQR)	3 (2; 5)	3 (1; 3.75)	3 (2; 5)	2 (2; 3.75)	0.52	0.72
History of travel**	78 (24.4)	1 (6.7)	77 (25.3)	22 (68.8)	0.10	**0.0001**
Household contact with a relative with confirmed COVID-19**	43 (13.5)	15 (100)	28 (9.2)	1 (3.1)	** < 0.0001**	** < 0.0001**
Comorbidities	49 (14.7)	1 (6.7)	48 (15.8)	7 (21.9)	0.33	0.20
*Clinical symptoms (observed at admission and in the course of the disease)*						
Fever	221 (69.3)	7 (46.7)	214 (70.3)	31 (96.8)	0.05	**0.0001**
Cough	223 (69.9)	6 (40.0)	217 (71.3)	27 (84.3)	**0.009**	**0.002**
Shortness of breath	24 (7.5)	1 (6.7)	23 (7.5)	3 (9.3)	0.89	0.75
Diarrhea	23 (7.2)	3 (20.0)	20 (6.5)	0	0.05	**0.009**
Vomiting	36 (11.3)	2 (13.3)	34 (11.1)	3 (9.3)	0.79	0.68
Rhinitis	60 (18.8)	1 (6.7)	59 (19.4)	8 (25.0)	0.06	0.14
Abdominal pain	10 (3.1)	0	10 (3.2)	0	0.47	-
Sore throat	33 (10.3)	0	33 (10.8)	8 (25.0)	0.17	**0.03**
Headache	15 (4.7)	0	15 (4.9)	3 (9.3)	0.37	0.22
Myalgia	25 (7.8)	0	25 (8.2)	3 (9.3)	0.24	0.22
Chest pain	12 (3.7)	0	12 (3.9)	2 (6.2)	0.43	0.32
Fatigue	14 (4.3)	0	14 (4.6)	3 (9.3)	0.39	0.22
Conjunctivitis	5 (1.5)	0	5 (1.6)	0	0.61	-
Skin rash	1 (0.3)	0	1 (0.3)	0	0.82	-
Asymptomatic	27 (8.4)	5 (33.3)	22 (7.2)	0	**0.0004**	**0.0006**
*Management and treatment*						
Hospitalization	125 (39.1)	11 (73.3)	113 (37.1)	26 (81.2)	**0.005**	0.54
PICU	0	0	0	0	-	-
Antibiotic therapy	94 (29.4)	7 (46.7)	87 (28.6)	5 (15.6)	0.13	**0.02**
Antiviral treatment (neuraminidase inhibitor)	30 (9.4)	1 (6.7)	29 (9.5)	30 (93.7)	0.71	** < 0.0001**
Chloroquine	1 (0.3)	1 (6.7)	0	0	-	-
Oxygen therapy	6 (1.8)	0	6 (1.9)	1 (3.1)	0.58	0.57
Mechanical ventilation	0	0	0	0	-	-
Corticosteroids	0	0	0	0	-	-

*P*-values set in bold indicate statistical significance (<0.05)

Data are presented as the number of patients with symptoms (%), unless otherwise indicated; *IQR* interquartile range, *PICU* pediatric intensive care unit.

*one patient with COVID-19/Influenza coinfection was excluded from this analysis.

**within 14 days before the onset of the disease.

**Figure 1:**
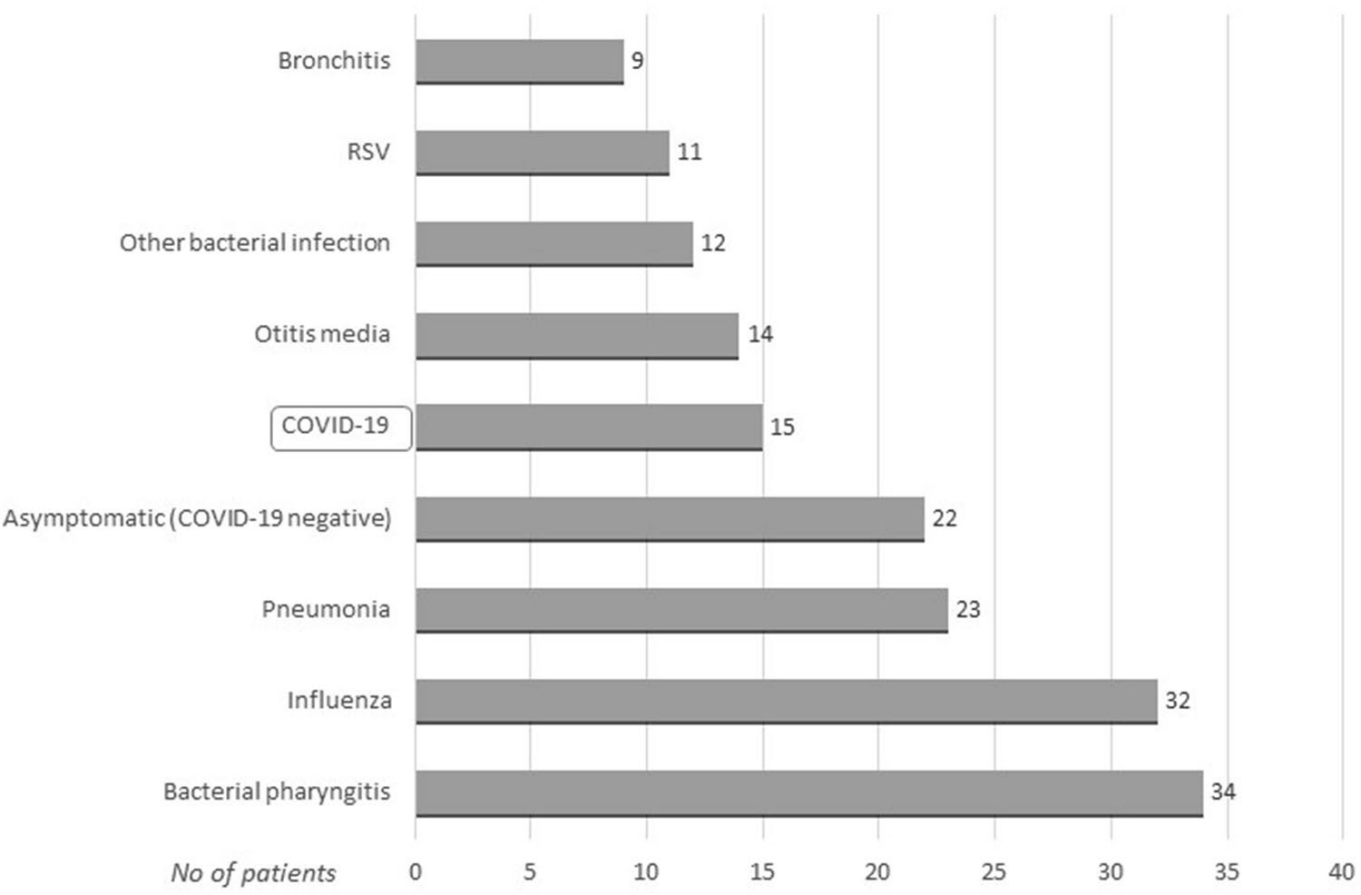
Final diagnoses in the study group (data available for 172 patients).

COVID-19 was confirmed in 15/319 (4.7%) of cases. Other diagnoses included bacterial pharyngitis, influenza, pneumonia, otitis media, RSV, bronchitis, and bacterial infections (e.g., urinary tract infection, gastrointestinal infections, bacteremia) (Fig. 1).

### Clinical characteristics of COVID-19 in children

Among SARS-CoV-2-infected children, there were 8 boys and 7 girls, with a mean age of 10.5 years (Table 1). One patient had an underlying disease (asthma). All of the children diagnosed with COVID-19 had close, household contact with an infected family member. Among our patients, there were two pairs of siblings. Only one child had a history of international travel within the 14 days before the onset of the disease; however, all our cases were diagnosed after local transmission in Poland had occurred (March 11, 2020).

**Figure 2:**
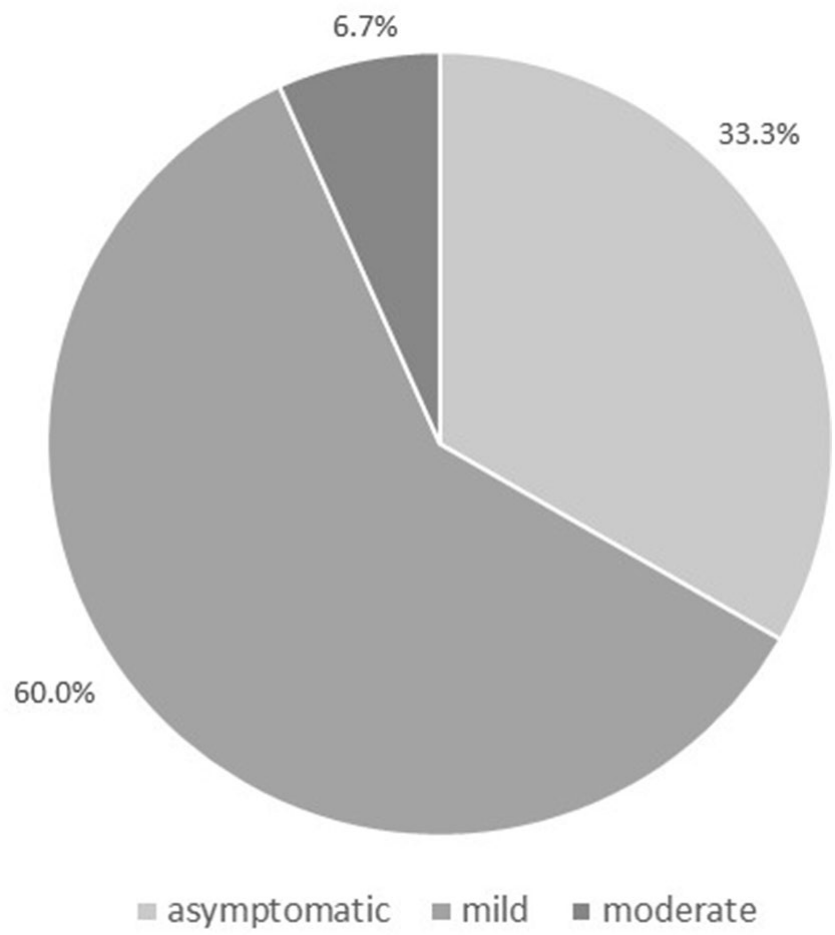
Clinical course of COVID-19 in the study group (n = 15). No child presented with a severe or critical course of the disease.

Five patients (33.3%) were asymptomatic and were tested for SARS-CoV-2 only because of household contact with an infected relative. In the remaining 10/15 (66.7%) cases, the course of the disease was symptomatic but mild-to-moderate (Fig. 2). The most commonly observed symptoms included fever, dry cough, diarrhea, vomiting, rhinitis, and shortness of breath (Table 1). However, the most frequently observed symptom—fever—occurred in only 46.7% of patients. Eleven children required hospitalization; however, in 7 cases, only a short 1-day hospitalization was necessary to perform clinical evaluation, laboratory testing, and chest X-ray, which was performed in 10 patients. In one case, radiological features of interstitial pneumonia were observed, and the clinical course of the disease was moderate. This patient received combined treatment with azithromycin and chloroquine. In one child, coinfection with influenza was diagnosed, and the patient received oseltamivir. In total, 7 symptomatic patients were treated with azithromycin according to local recommendations that indicate the immunomodulatory influence of this substance. In two patients, bacterial superinfection was diagnosed. These patients received beta-lactam antibiotics. Severe complications of the disease were not observed in any of the patients. None of the patients qualified for treatment in the pediatric intensive care unit (PICU), and no child required oxygen therapy or mechanical ventilation. Thus, in no case was the clinical course of the disease described as severe or critical.

### Comparison between COVID-19-positive and COVID-19-negative patients

In comparison with other patients from the study group, significantly more children with COVID-19 had contact with an infected family member (100% vs. 9.2%, *P* < 0001). An analysis of clinical presentation data showed that cough was significantly less frequent in the COVID-19-positive group (40.0% vs. 84.3%, *P* = 0.009). In addition, there was a trend towards less frequent fever and rhinitis (46.7% vs. 70.3%, *P* = 0.05; 6.7% vs. 19.4%, *P* = 0.06, respectively), as well as more frequent diarrhea in children with COVID-19 (20.0% vs. 6.5%, *P* = 0.05). Compared with COVID-19-negative patients, infected children were more commonly hospitalized (73.3% vs. 37.1%, *P* = 0.005); however, a higher proportion of them were asymptomatic (33.3% vs. 7.2%, *P* = 0.0004).

### Comparison between COVID-19 and influenza

Influenza was one of the most commonly diagnosed diseases in our study group (32/319, 10%) and was more than twice as common as COVID-19 (4.7%). Comparison between COVID-19 and influenza patients revealed a more frequent history of household contact with a SARS-CoV-2-infected person among COVID-19 patients (100% vs. 3.1%, *P* < 0.0001). In contrast, influenza patients had significantly more frequent travel abroad (68.8% vs. 6.7%, *P* = 0.0001). The most popular destination was Italy (15/22, 68.2%), and the remaining patients travelled to United Kingdom (2), Germany (2), France (1), Spain (1), and Austria (1). Clinical presentation of the diseases differed significantly. First, there were no asymptomatic cases of influenza compared to 33.3% of cases with asymptomatic COVID-19 (*P* = 0.0006). In symptomatic patients, the frequency of fever, cough and sore throat was significantly higher in the influenza group (96.8% vs. 46.7%, *P* = 0.0001; 84.3% vs. 40.0%, *P* = 0.002; 25.0% vs. 0, *P* = 0.03, respectively), whereas COVID-19 patients more frequently suffered from diarrhea (20.0% vs. 0, *P* = 0.009). The groups did not differ in the proportion of hospitalized patients; however, antibiotic treatment was more commonly implemented in the COVID-19 group (46.7% vs. 15.6%, *P* = 0.02).

### Role of epidemiological and clinical evaluation for predicting COVID-19 diagnosis

Our observations revealed that the highest risk of infection exists when the presence of clinical symptoms is accompanied by confirmed contact with an infected family member (62.5%). The risk was lowest when the epidemiological history was negative, even in the presence of the clinical symptoms (0%, Table 2).

**Table 2:** Assessment of the risk of COVID-19 according to epidemiological data and the presence of clinical symptoms.

Household contact with a relative with COVID-19	Presence of the typical clinical symptoms	Total	COVID-19 positive	COVID-19 negative	*P*
+	+	16	10 (62.5)	6 (37.5)	< 0.0001
+	−	27	5 (18.5)	22 (81.5)	
−	+	276	0	276 (100)	

Data are presented as numbers (%). In our group, there were no asymptomatic children without any contact with an infected person.

## Discussion

COVID-19 is a novel infection and has been known for only a few months. Thus, knowledge of the disease, especially in specific groups of patients, e.g., children, is scarce and limited. As the epidemic is ongoing, every reported observation on the epidemiological and clinical characteristics of the infection is essential for our understanding of the disease. To date, a large share of the scientific evidence has originated in China. It is possible that country-specific factors (e.g., nutrition, epidemiological influences, day care) in European children may differ from the Chinese population^14^. To the best of our knowledge, we present one of the first reports on COVID-19 in the pediatric population outside of China.

In our group of 319 patients suspected for COVID-19, 4.7% were positive. In a study performed in Madrid by Tagarro et al., 41 of 365 (11.2%) pediatric patients had positive test results during the first two weeks of the epidemic in Spain^4^. In early January 2020, in Wuhan, of 366 children screened for SARS-CoV-2 infection, 6 patients (1.6%) were positive^15^. The differences in these cohorts may result from different indications for testing, *e.g.,* testing all suspected cases or only symptomatic patients. Since children are often asymptomatic, this may significantly influence the proportion of the positive results. The median age of our patients was 10.5 years (range 10 months–14 years). In other studies, the mean age of affected children differed. In the study from Madrid, the median age was 3 years (range 0–15 years); in the US, among all 2572 COVID cases, the median age was 11 years (range 0–17)^4,7^. In the largest Chinese pediatric case series by Dong et al. that reported 2143 patients with COVID-19, the mean age was 7 years, similar to the study from Wuhan on 171 patients, in which the median age was 6.7 years (1 day–15 years)^8,9^. No significant predominance of sex was found in any pediatric report. In the study by Dong et al., there were 56.6% boys in the study group, which is similar to our 53.3%^9^.

Available data suggest that the main source of infection in children is household exposure, as 56.0%—90% of the diagnosed children had an infected family member^1,6^. This trend was even more pronounced in our study, where family clustering occurred for all infected children. In addition, among our patients, there were two pairs of siblings. Our observations revealed that the highest risk of infection exists when the presence of clinical symptoms is accompanied by confirmed contact with an infected family member (62.5%).

On the basis of previously published data, COVID-19 symptoms seem to be less severe in children than in adults^9,14^. Approximately 10% of cases in children are asymptomatic^1^. In the study by Dong et al., with the largest child case series so far, over 90% of the 2143 patients diagnosed with COVID-19 had either asymptomatic or mild-to-moderate disease^9^. In the remaining 5.2%, the course of the disease was severe, and in 0.6%, it was critical^9^. In our group, 33.3% patients were asymptomatic, which may result from the fact that we tested asymptomatic children with confirmed contact with an infected relative. In the remaining patients, the course of the disease was mild-to-moderate, with no severe or critical cases. Several explanations for the milder presentation of COVID-19 in children have been suggested^14,16^. First, children might have a different immune response to SARS-CoV-2 than adults^16^. Children, especially young children, tend to have repeated exposure to many viral infections, which may benefit their immune system when it responds to SARS-CoV-2^14^. Second, the presence of other viruses in the mucosa of the airways, which is common in children, may limit the growth of SARS-CoV-2 by direct virus-to-virus competition^16^. Another possibility is that the S protein of SARS-CoV-2 binds to angiotensin-converting enzyme 2, which is less mature in young children, protecting them against the virus^14^.

Observations from the US on 291 pediatric and 10,944 adult patients revealed that clinical symptoms of COVID-19 are observed less frequently in children than in adults^7^. A previous report found that 73% of children and 93% of adult patients had symptoms of fever, cough, or shortness of breath^7^. According to Chinese reports on pediatric COVID-19 patients, the most common symptoms were fever, which occurred in 44–50% of children, and cough, experienced by 38% of patients, followed by rhinitis, fatigue, headache, diarrhea, and dyspnea^1,17,18^. This is similar to our observations that fever occurred in 46.7% and cough in 40% of the COVID-19 patients. Interestingly, both symptoms were significantly more frequent in patients negative for COVID-19 (70.3% and 71.3%, respectively). In addition, gastrointestinal symptoms were observed more commonly in the COVID-19-positive patients: 20% experienced diarrhea and 13.3% experienced vomiting. In the US cohort, these symptoms were observed in 13% and 11% of children, respectively^7^. In a study that included 171 children from Wuhan Children’s Hospital, diarrhea occurred in 8.8% of patients, and vomiting occurred in 6.4% of patients^8^. These observations suggest, that gastrointestinal symptoms are common in children infected with SARS-CoV-2 and they should trigger tests for COVID-19.

Eleven children in our group (73.3%) were hospitalized. This proportion is higher compared to other cohorts, e.g., the US, where 1.6–2.5% of 123 patients required hospitalization^19^. However, in 7 out of 11 cases, only a short 1-day hospitalization was necessary to perform clinical evaluation, laboratory testing and chest X-ray. In one patient, radiological features of pneumonia were observed. This patient received combined treatment with azithromycin and chloroquine as part of a clinical trial. The child recovered without severe complications. No severe or critical cases requiring hospitalization in the PICU were observed in our group, similar to observations of other authors from China^6,18^. However, several individual cases of children requiring mechanical ventilation and PICU admission have been reported thus far^4,14^. With an increasing prevalence of COVID-19 during the next waves of pandemic, asymptomatic children or those with a mild course of the disease, would not require hospitalization due to the SARS-CoV-2 infection. Children presenting with more severe course of COVID-19, in particular when oxygen therapy is needed, or patients with comorbidities, should be referred to the hospital.

A significant number of our patients were negative for COVID-19, and other diagnoses were established, including bacterial infections, that required proper treatment. This is an essential finding during a pandemic, when access to health services may be limited. In symptomatic children suspected of having COVID-19, other, more common infections are possible and should not be overlooked. To our knowledge, there are no published data that compare the clinical courses of COVID-19 and influenza in children. In our study, 10% of patients suspected of having COVID-19 were infected with influenza virus. This rate is similar to observations from Wuhan, where among 366 children, influenza A or B was detected in 43 (11.8%) patients^15^. In one patient, coinfection with COVID-19 and influenza was diagnosed. Clinical presentation of both diseases differed significantly. First, all cases of influenza were symptomatic and had a higher frequency of fever, cough, and sore throat, whereas COVID-19 patients more frequently suffered from diarrhea. There was no difference between groups in the proportion of hospitalized patients; however, antibiotic treatment was more commonly implemented in the COVID-19 group.

This study was limited by a small number of children with a confirmed SARS-CoV-2 infection. We did not include asymptomatic patients with no epidemiological history, which might have influenced the final number of infected cases. However, considering that the pandemic is ongoing, the results presented here provide valuable data for understanding the epidemiological and clinical features of COVID-19 in the pediatric population.

On the basis of our experience, we conclude that the clinical course of COVID-19 in the pediatric population is usually mild or asymptomatic. In symptomatic children with suspected COVID-19 and those who have been screened for COVID-19, other infections are common and should not be overlooked. The main risk factor for SARS-CoV-2 infection in children is close household contact with an infected relative. Thus, children who have an infected family member should be tested for COVID-19, irrespective of their clinical presentation.

## Data Availability

The datasets used and analyzed during the current study are available from the corresponding author upon reasonable request.
